# Correction: Characterizing Alzheimer’s Disease Severity via Resting-Awake EEG Amplitude Modulation Analysis

**DOI:** 10.1371/journal.pone.0101613

**Published:** 2014-06-24

**Authors:** The PLOS ONE Staff

## Notice of Republication

This article was republished on June 6, 2014, to correct missing text in the article. The publisher apologizes for the error. Please download this article again to view the correct version. The originally published, uncorrected article and the republished, corrected article are provided here for reference.

## Supporting Information

File S1 Originally published, uncorrected article.
(PDF)

File S2 Republished, corrected article.
(PDF)
