# Supplementary material for: Correction: Characterizing Alzheimer’s Disease Severity via Resting-Awake EEG Amplitude Modulation Analysis
Source: PLoS One. 2014 Jun 24;9(6):e101613. doi: 10.1371/journal.pone.0101613 (PMC4069187; doi:10.1371/journal.pone.0101613)
Supplement: File S1 — Originally published, uncorrected article. (PDF) [file pone.0101613.s001.pdf]

PLOS ONE

# Characterizing Alzheimer's Disease Severity via Resting-Awake EEG Amplitude Modulation Analysis

**Francisco J. Fraga[1,2], Tiago H. Falk[1]*, Paulo A. M. Kanda[3], Renato Anghinah[3]**

**1** Institut National de la Recherche Scientifique (INRS-EMT), University of Quebec, Montreal, Quebec, Canada, **2** Engineering, Modelling and Applied Social Sciences Center, Universidade Federal do ABC, Santo André, São Paulo, Brazil, **3** Reference Center of Behavioural Disturbances and Dementia, School of Medicine, Universidade de São Paulo, São Paulo, Brazil

## Abstract

Changes in electroencephalography (EEG) amplitude modulations have recently been linked with early-stage Alzheimer's disease (AD). Existing tools available to perform such analysis (e.g., detrended fluctuation analysis), however, provide limited gains in discriminability power over traditional spectral based EEG analysis. In this paper, we explore the use of an innovative EEG amplitude modulation analysis technique based on spectro-temporal signal processing. More specifically, full-band EEG signals are first decomposed into the five well-known frequency bands and the envelopes are then extracted via a Hilbert transform. Each of the five envelopes are further decomposed into four so-called modulation bands, which were chosen to coincide with the delta, theta, alpha and beta frequency bands. Experiments on a resting-awake EEG dataset collected from 76 participants (27 healthy controls, 27 diagnosed with mild-AD, and 22 with moderate-AD) showed significant differences in amplitude modulations between the three groups. Most notably, i) delta modulation of the beta frequency band disappeared with an increase in disease severity (from mild to moderate AD), ii) delta modulation of the theta band appeared with an increase in severity, and iii) delta modulation of the beta frequency band showed to be a reliable discriminant feature between healthy controls and mild-AD patients. Taken together, it is hoped that the developed tool can be used to assist clinicians not only with early detection of Alzheimer's disease, but also to monitor its progression.

**Citation:** Fraga FJ, Falk TH, Kanda PAM, Anghinah R (2013) Characterizing Alzheimer's Disease Severity via Resting-Awake EEG Amplitude Modulation Analysis. PLoS ONE 8(8): e72240. doi:10.1371/journal.pone.0072240

**Editor:** Eshel Ben-Jacob, Tel Aviv University, Israel

**Received** May 6, 2013; **Accepted** July 8, 2013; **Published** August 27, 2013

**Copyright:** © 2013 Fraga et al. This is an open-access article distributed under the terms of the Creative Commons Attribution License, which permits unrestricted use, distribution, and reproduction in any medium, provided the original author and source are credited.

**Funding:** This work was funded by the Natural Sciences and Engineering Research Council of Canada (NSERC) and the Foundation for Research Support of the State of São Paulo (FAPESP). The funders had no role in study design, data collection and analysis, decision to publish, or preparation of the manuscript.

**Competing Interests:** The authors have declared that no competing interests exist.

* E-mail: falk@emt.inrs.ca

## Introduction

In North America, Alzheimer's disease (AD) amounts to 60–80% of reported dementia cases [1]. Over the last decade, AD has become the fifth leading cause of death in North Americans aged over 65 years, with an increase in death rate of 66% between 2000 and 2008 [2]. Moreover, recent reports have shown the costs with dementia surpassing those of heart disease and cancer [3]. In 2012, the World Health Organization and Alzheimer's Disease International released a report calling on governments to implement national dementia plans focusing on 1) raising public awareness about the disease and reducing stigma, 2) improving early diagnosis, and 3) providing better care and more support to caregivers [4]. Here, special emphasis is placed on the second focus area - early diagnostics - as it is critical in order to initiate treatment that can significantly retard disease progression, thus potentially leading to improved patient quality of life, reduced caregiver stress, and lower health care expenditures [5]. Currently, diagnosis of AD may be done via neuropsychological evaluations which require lengthy experimental sessions and experienced professionals. Definite diagnosis, however, can only be established with a histopathological analysis of the brain [6]. It is clear that improved *objective* detection methods are still needed.

Neuroimaging techniques, such as computerized tomography (e.g., [7]), magnetic resonance imaging (e.g., [8]), and positron emission tomography (e.g., [9]) have emerged as promising tools to assist clinicians with early diagnosis of AD by detecting visible structural and functional changes in the brain [10]. Magneto- (MEG) and electro-encephalography (EEG), which directly reflect functional and anatomical changes in the cerebral cortex, have also emerged as a prominent candidate for AD diagnosis [11], with diagnostic sensitivity and specificity in line with more complex neuroimaging techniques [12]. Traditionally, two signal analysis methods have been employed for AD diagnosis, namely spectral (e.g., [13–15]) and nonlinear dynamics (e.g., [16,17]), with studies showing a direct link between the two approaches [18].

More recently, amplitude modulation analysis of neuronal oscillations at rest has also emerged as a promising tool to characterize different neurological disorders (e.g., [19,20]). The authors in [21], for example, showed the importance of amplitude modulation of neuronal oscillations for encoding and retention of information in memory. It was found that impairments in the coordination of oscillatory activity were present with AD based on detrended fluctuation analysis (DFA) of MEG amplitude envelopes. While DFA has been shown to be useful for EEG/MEG analysis [20], studies have suggested that the discriminatory power obtained with DFA is in line with that obtained via traditional spectral analysis [22]. Recently, an alternate spectro-temporal analysis technique was proposed to characterize EEG amplitude modulation changes in patients with moderate-stage AD [23], with improved discriminability over conventional spectral analysis [24].

Here, we build upon the work of [23] and hypothesize that EEG amplitude modulation spectro-temporal dynamics can serve not only as a discriminatory feature between healthy aging and early-stage AD, but also as a useful feature to characterize the severity of the disease. Our experimental results support this hypothesis.

## Materials and Methods

### Ethics Statement

Ethics approval was obtained from the School of Medicine, São Paulo University. All recruited participants provided written consent.

### Participants

Seventy six participants were recruited through the Reference Center of Behavioral Disturbances and Dementia at the Clinical Hospital, School of Medicine, São Paulo University. AD diagnosis was made according to NINCDS-ADRDA [25] and DSM-IV-TR [26] criteria and disease progression classification was based on the Brazilian version of the Mini-Mental State Examination (MMSE) [27] and the Clinical Dementia Rating (CDR) scale. These 76 participants were separated into three age-matched groups, namely NS, AD1 and AD2. Group NS comprised 27 healthy older adults (age: , 15 female), group AD1 was composed by 27 mild-AD patients (, 16 female) and the third group (AD2) included 22 patients with moderate AD (, 15 female). All subjects from the NS group had , for the AD1 group the inclusion criterion was , and the AD2 group only included patients with . The mean MMSE scores of three cohorts were significantly different at and , respectively. The two AD cohorts were education matched (AD1: years; AD2: years) but the healthy controls had a significantly higher level of education (NS: years). Participants had no history of diabetes mellitus, kidney diseases, thyroid diseases, alcoholism, liver disease, lung disease, or vitamin B12 deficiency, factors which could also lead to cognitive impairment.

### Data Collection and Pre-processing

Twenty-channel EEG signals were collected using the *Braintech 3.0* instrumentation (EMSA Equipamentos Médicos Inc., Brazil), digitized with a 12-bit analog-to-digital converter and sampled at a rate of 200 Hz; impedance was maintained below 10 k. Placement of scalp electrodes (referential montage) followed the international 10–20 system. Linked-ear referential electrodes (A1 and A2) were used, as recommended by the Brazilian Society of Clinical Neurophysiology and the American EEG Society. During examination, EEG was recorded with the participants awake and resting with their eyes closed. An infinite impulse response low-pass elliptic filter with a zero at 60 Hz was applied to eliminate any power grid interference. For each participant, between 28 and 40 eight-second epochs (mean 37.97, sd 3.86) were selected per EEG channel by experienced physicians. The selected epochs were free of eye movement, electromyographic activity, and head motion artifacts. Furthermore, to ameliorate the effects of different amplifier/impedance settings between channels and participants, EEG epoch data for each channel was normalized by the total EEG power present in the given channel.

### EEG Spectro-temporal Amplitude Modulation Analysis

Figure 1 depicts the signal processing steps involved in the spectro-temporal EEG amplitude modulation analysis technique. First, the full-band EEG signal is decomposed into five sub-bands, well-known in the literature as the delta (Hz), theta (Hz), alpha (Hz), beta (Hz), and gamma (Hz) bands [28]. The temporal envelope of each of the five sub-band EEG signals is then computed by means of a Hilbert transform [29]. The subplots on the right of Fig. 1 illustrate representative EEG sub-band signals (gray) and their respective Hilbert amplitude envelopes (black). In order to quantify the temporal dynamics of the sub-band envelopes, we perform a second frequency decomposition into five so-called modulation bands using second-order bandpass modulation filters (with quality factor ). The resulting frequency-frequency signal representation conveys rate-of-change information of each of the five sub-band envelopes.

In order to explore possible cross-frequency modulation interaction, the ranges of the modulation frequency bands were empirically designed to coincide with the ranges of the conventional frequency bands. Cross-frequency amplitude modulation interaction is an under explored area that may lead to insights into complex temporal pattern changes with different stages of AD [30]. To distinguish between modulation and frequency bands, we will refer to the former as m-delta (Hz), m-theta (Hz), m-alpha (Hz), m-beta (Hz), and m-gamma (Hz) throughout the remainder of this paper. From properties of the Hilbert transform and following Bedrosian's theorem, the envelope signal can only contain frequencies (i.e., modulation frequencies) up to the bandwidth of its originating signal [31,32]. Having this said, it only makes sense to compute cross-frequency amplitude modulation interaction (here represented by "E(frequency band; modulation band)," the percentage of modulation energy present in a given frequency and modulation band relative to the energy across all bands) for the following scenarios: E(delta; m-delta), E(theta; m-delta, m-theta), E(alpha; m-delta, m-theta), E(beta; m-delta, m-theta, m-alpha, m-beta), and E(gamma; m-delta, m-theta, m-alpha, m-beta, m-gamma). In our experiments, these 14 percentage cross-frequency modulation parameters are computed for each of the 19 EEG channels. The interested reader is referred to [23] for a more detailed description of the signal processing steps involved.

### EEG Power Spectrum Analysis: Benchmark

A prominent change reported in the literature between AD and healthy controls is that of 'EEG slowing'. This slowing is commonly measured as an increase in EEG power in the delta and theta frequency bands and a decrease in spectral power in the alpha and beta bands, particularly in the occipital and temporo-parietal regions [13,33–37]. In order to benchmark, as well as test the complementarity of the proposed spectro-temporal features, a conventional EEG power spectrum analysis was also performed across the five well-known frequency bands.

### Statistical and Correlation Analyses

Statistical significance was established at 1% level for all tests. Normality was verified for all parameters using a Jarque-Bera test [38] with critical values computed by Monte-Carlo simulation [39]. For normal distributed features, one-way ANOVA with parameters across the three groups at different locations on the scalp was used. A non-parametric Kruskal-Wallis test was applied when the assumption of normal distribution was not confirmed for a given feature. In either case, a Dunn-Sidak post-hoc test was used for multiple comparisons correction. Additionally, estimates of reliability were obtained using split-half Pearson correlations. In this reliability test, epochs from each participant were separated into two disjoint subsets assuring that no temporal overlap existed between the epochs from each subset, thus consequently assuring statistical independence between the observations. Pearson correlations were then computed between the two subsets; this was done separately for the healthy participants, and mild- and moderate-AD patients.

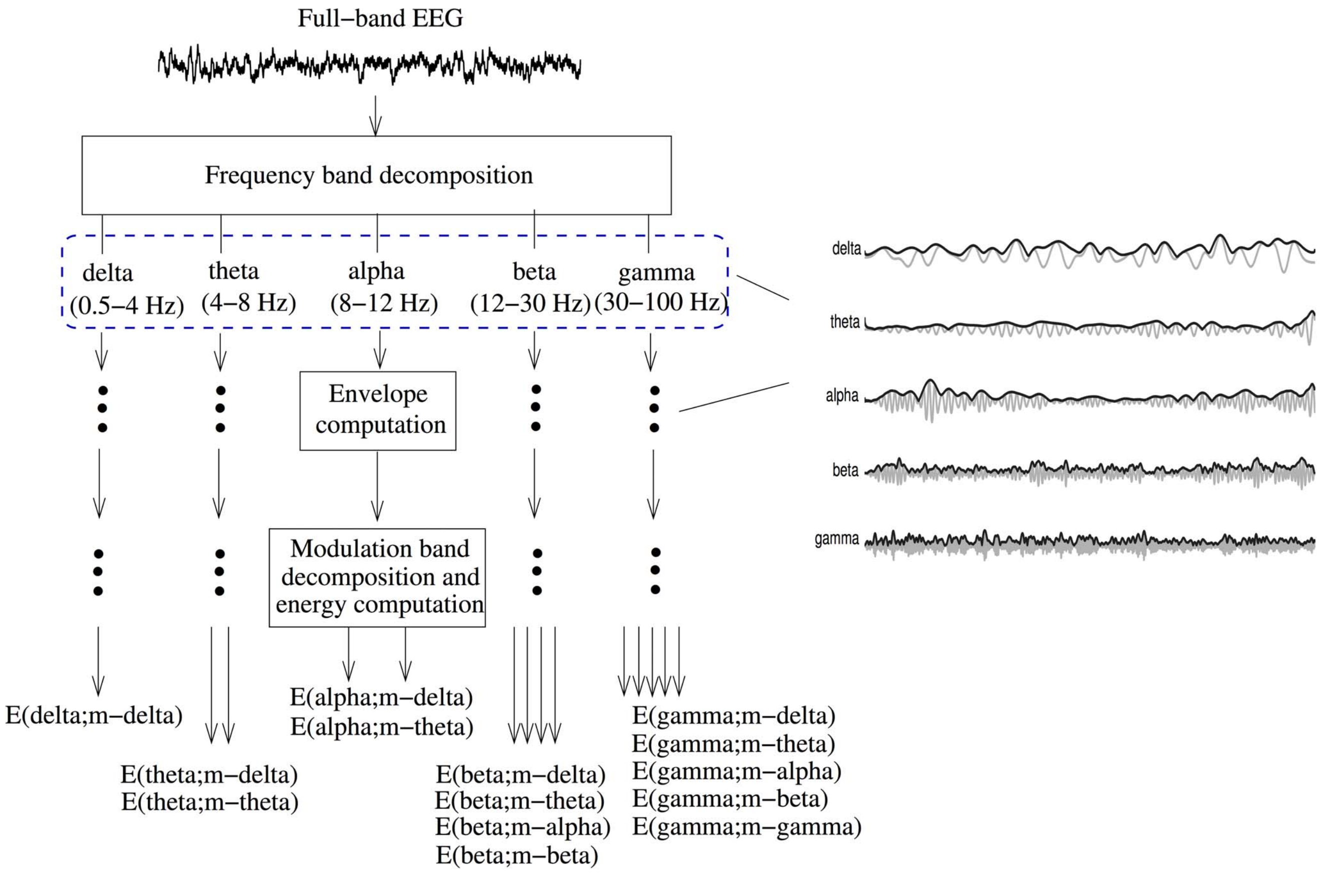


**Figure 1. Signal processing steps used to compute resting EEG spectro-temporal modulation energy.** Subplots on the right illustrate the five sub-band EEG signals (gray) and their respective Hilbert temporal envelopes (black).
doi:10.1371/journal.pone.0072240.g001

## Results

Tables 1 and 2 show all the locations, frequency bands, and modulation frequencies in which significant increases (↑) or decreases (↓) were observed ($p<0.01$) for the AD1 vs. NS and AD2 vs. AD1 post-hoc comparisons, respectively. As can be seen, for the AD1 vs. NS post-hoc pairwise comparison, an EEG amplitude modulation energy decrease in the high-frequency alpha and beta bands was observed with the AD1 group. While this significant decrease was only observed for the Pz location for the alpha frequency band, several temporal, frontal, occipital and parietal locations were found for the beta band. Alternately, an increase in low-frequency delta and theta band modulation energy was observed with AD1, particularly in the temporal, parietal, and occipital regions. For the AD2 vs. AD1 comparison, significant increases in theta band m-delta and m-theta modulations were found with AD2 over all tested locations. In turn, significant decreases in modulation energy in the beta band were found in the temporal, central, parietal, and occipital regions. In both pairwise comparisons, features extracted from the gamma frequency band were not shown to be significantly different. Figures 2 and 3 depict the average topographical maps of the EEG m-delta amplitude modulation ratio parameters for the NS, AD1 and AD2 groups for the theta and beta frequency bands, respectively.

In order to benchmark the obtained results, post-hoc pairwise comparisons of EEG powers were also performed. Corroborating results reported in the literature (e.g., [13]), for the AD1 vs. NS comparison we found 1) a significant increase in delta band power in positions O1 and Pz; 2) a significant increase in theta band power in select positions in the occipital and parietal regions (O1/O2, P4); 3) a significant decrease in alpha band power in position Pz; and 4) a significant decrease in beta band power in positions F8, Oz, P4, and T5/T6. On the other hand, for the AD2 vs. AD1 comparison, we found 1) a significant increase only in the theta band (across the majority of the electrode positions), and 2) a significant decrease only in the beta band, at positions C3, Cz, P3 and T3.

Relative to Table 1, it can be seen that for the theta and alpha bands, significant differences are observed in the same positions for both methods, suggesting that not only are overall powers different, but also how they are modulated over time. For the delta and beta bands, on the other hand, significant differences can be detected across an increased number of brain regions, thus suggesting a potential complementarity of the proposed features for discriminating between healthy controls and mild-AD. Similarly, for the AD2 vs AD1 comparison with Table 2, both methods were effective in finding significant differences between the two groups across the majority of the electrode positions for the theta band. For the beta band, however, the conventional power spectrum analysis resulted in significant differences only in the midline and left hemisphere. With the proposed features, however, significant differences arise also in the right hemisphere and the occipital regions. Taken together, these findings suggest that the proposed spectro-temporal amplitude modulation analysis may provide a richer, and perhaps complementary, pool of data for automated EEG-based AD diagnosis.

**Table 1.** Electrode location and frequency bands at which mild-AD patients present significantly ($p < 0.01$) greater ( ↑ ) or lower ( ↓ ) modulation spectral energy than normal elderly subjects.

| Electrode | Delta | Theta | | Alpha | | Beta | | | |
|---|---|---|---|---|---|---|---|---|---|
| position | m-delta | m-delta | m-theta | m-delta | m-theta | m-delta | m-theta | m-alpha | m-beta |
| F8 | | | | | | ↓ | ↓ | | ↓ |
| T5 | ↑ | | | | | ↓ | | | |
| T6 | | | | | | ↓ | ↓ | | |
| Pz | ↑ | | | ↓ | ↓ | | | | |
| P4 | ↑ | | ↑ | | | ↓ | ↓ | | |
| O1 | ↑ | ↑ | | | | ↓ | | | |
| Oz | | | | | | ↓ | ↓ | ↓ | ↓ |
| O2 | ↑ | ↑ | ↑ | | | ↓ | | | |

Frequency bands which did not result in significant differences are not shown for brevity.
doi:10.1371/journal.pone.0072240.t001

Lastly, to gauge the reliability of the proposed measure, split-half Pearson correlations were computed for each post-hoc comparison test. For the AD1 vs. NS test, a split-half correlation of and was obtained for the NS and AD1 groups, respectively. For the AD2 vs. AD1 test, a split-half correlation of and was obtained for AD1 and AD2 groups, respectively. As can be seen, reliability is high and consistent across channels, frequency bands, and EEG amplitude modulation frequencies for the three groups.

**Table 2.** Electrode location and frequency bands at which moderate-AD patients present significantly ($p < 0.01$) greater ( ↑ ) or lower ( ↓ ) modulation spectral energy than those with mild-AD.

| Electrode | Theta | | Beta | | |
|---|---|---|---|---|---|
| position | m-delta | m-theta | m-theta | m-alpha | m-beta |
| Fp1 | ↑ | ↑ | | | |
| Fp2 | ↑ | ↑ | | | |
| F7 | ↑ | ↑ | | | |
| F3 | ↑ | ↑ | | | |
| Fz | ↑ | ↑ | | | |
| F4 | ↑ | ↑ | | | |
| T3 | ↑ | ↑ | ↓ | ↓ | ↓ |
| T4 | ↑ | ↑ | | ↓ | |
| T5 | ↑ | ↑ | | ↓ | ↓ |
| T6 | ↑ | ↑ | | | |
| C3 | ↑ | ↑ | ↓ | ↓ | ↓ |
| Cz | ↑ | ↑ | ↓ | ↓ | ↓ |
| C4 | ↑ | ↑ | | ↓ | |
| P3 | ↑ | ↑ | ↓ | ↓ | |
| Pz | ↑ | ↑ | | ↓ | |
| P4 | ↑ | ↑ | | | |
| O1 | ↑ | ↑ | | | |
| O2 | ↑ | ↑ | ↓ | ↓ | ↓ |

Frequency bands which did not result in significant differences are not shown for brevity.
doi:10.1371/journal.pone.0072240.t002

## Discussion

### Characterizing Mild-Stage AD

Previous EEG studies have shown significant increases and decreases in theta and alpha spectral powers, respectively, in mild-stage AD relative to healthy controls (e.g., [13,36]). Here, we have shown that not only are the aforementioned frequency band powers affected (particularly in occipital and parietal regions), but also how their envelopes are modulated over time, thus potentially providing complementary information over traditional spectral insights, as shown by [24]. In relationship to the DFA-based amplitude analysis proposed by [21], we have also found decreased alpha modulation in parietal regions but did not find

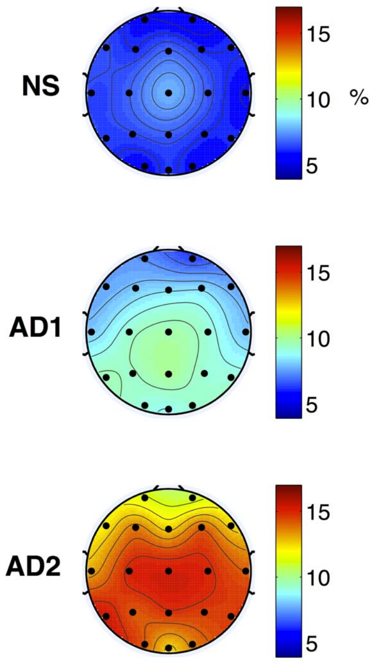


**Figure 2. Topographical maps of average NS (top), AD1 (middle), and AD2 (bottom) modulation frequency responses.** Plots represent the m-delta modulation frequency content in the theta frequency band expressed as percentage over all five frequency and four modulation frequency bands.
doi:10.1371/journal.pone.0072240.g002

significant differences in prefrontal theta modulations. As such, further studies are needed to investigate the complementarity of the DFA and spectro-temporal based amplitude analysis techniques. Moreover, we have found significant increases in delta modulations of the delta frequency band across several temporal, parietal, and occipital regions. Previous literature, however, has shown an increase in delta band power [40] and a decrease in beta band power [13] only in late-stage AD, with only subtle a increase in delta band power seen with mild-AD in the occipital regions [36]. These findings suggest that changes in slow-wave envelope dynamics may be detectable at earlier stages of the disease, thus potentially assisting clinicians with earlier diagnostics.

## Characterizing Disease Progression

From Tables 1–2 and Figures 2–3, it can be seen that as disease severity progressed (AD1 to AD2), further increases in theta band amplitude modulations occurred across a wider network of brain regions. Moreover, a decrease in beta band amplitude modulation across temporal, central, parietal, and occipital regions was observed, thus corroborating previous findings on the shift of beta band power to more anterior positions as disease progresses [41]. Notwithstanding, previous literature has observed increases in theta band and decreases in beta band powers across all brain regions only in patients with severe AD (CDR = 3) [13]. Being able to characterize amplitude modulation changes at earlier stages of the disease may allow for quantitative treatment outcome measures to be developed, thus improving disease progression monitoring. Moreover, using conventional power spectrum analysis, it was found that most significant differences in beta power occurred in the left hemisphere, thus corroborating previous findings [37]. With the proposed spectro-temporal features, on the other hand, significant differences were found across the two hemispheres, with beta m-alpha differences, for example, occurring between inter-hemispheric mirror-image locations P3–P4 and C3–C4. Such findings may be linked to the inter-hemispheric disconnection previously-reported for AD [16].

## Characterizing Cross-Frequency Interactions: Some Hypotheses

An advantage of the used signal processing tool is its capability to characterize amplitude-amplitude cross-frequency interaction, thus potentially leading to greater insight into the changes in functional dynamics of brain systems with AD progression. For example, it was observed that beta rhythms were mostly modulated at a rate that coincides with the theta band frequency (see Table 2), with decreased interaction (lower modulation energy) as disease progressed (AD2 vs AD1). Interestingly, reduced beta-theta interaction has been attributed to lower reward-gain motivation [42], a behavioural and psychological symptom observed with disease progression [43].

Moreover, from Tables 1–2 and Fig. 3, it can be seen that a significant beta-delta interaction disappeared when comparing the AD2 vs AD1 groups relative to AD1 vs NS. Decoupling of beta-delta interaction has been associated with behavioural activation and fearlessness [44,45], a symptom associated with frontal lobe impairments commonly observed with late-stage AD [46]. On the other hand, existence of beta-delta interaction has been associated with anxiety [47,48], a prevalent neuropsychiatric symptom observed in patients with mild cognitive impairment and mild-AD [49,50], thus corroborating the findings presented in Table 1 for mild-AD patients. It can also be observed from the Tables that there was a significant drop in beta-alpha interaction (lower modulation energy) as the disease severity increased across multiple temporal, parietal, central, and occipital regions.

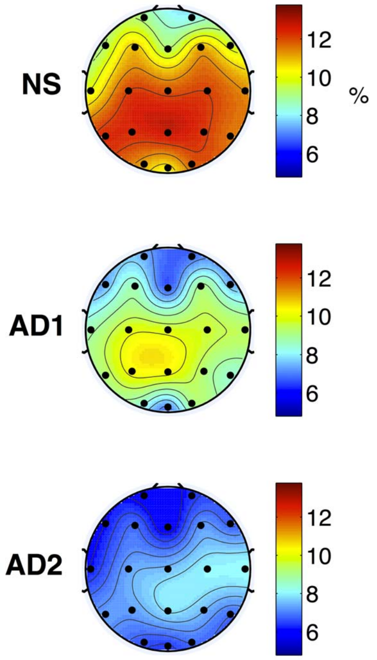


**Figure 3. Topographical maps of average NS (top), AD1 (middle), and AD2 (bottom) modulation frequency responses.** Plots represent the m-delta modulation frequency content in the beta frequency band expressed as percentage over all five frequency and four modulation frequency bands.
doi:10.1371/journal.pone.0072240.g003

Decreased beta-alpha *phase* coupling has been previously linked with lower cognitive ability [51], a symptom commonly observed and expected from AD and its progression. Further studies are needed to investigate if the obtained *amplitude* modulation interaction findings are also related to cognitive load.

Lastly, the most significant change observed from the AD1 vs NS and AD2 vs AD1 comparisons was with the theta-delta cross-frequency interactions (see Tables 1–2 and Fig. 2). In the former scenario, significant differences only occurred in the occipital regions, whereas in the latter, they occurred across almost all tested sites, spanning pre-frontal, frontal, temporal, central, parietal and occipital regions. In transgenic mouse models of Alzheimers disease, increased theta-delta interaction has been observed in non-rapid eye movement sleep EEG relative to control wild type mice, with more prominent increases occurring with age, i.e., directly related to increased amyloid beta deposition [52,53]. While a direct comparison with humans is not possible, this is an interesting finding as amyloid beta deposits can be found in abundance in the occipital cortex of demented individuals [54]. Further studies are needed to validate the relationship between theta-delta interaction and amyloid deposits, as such relationship may be useful in very early diagnosis of AD.

## Study Limitations

Findings reported here are based on a sample size of 76 participants, divided into three groups. Future studies should focus on a larger, more gender-balanced participant pool, as gender differences may also play a factor, as reported by [55]. Notwithstanding, a prevalence of AD within women has been previously reported [55,56]. Moreover, an effort was made to achieve education-matched groups; however, this could only be achieved with the AD1 and AD2 patient groups, thus corroborating the previously-reported link between AD and years of

schooling [56–58]. Lastly, while this study looked at differences between healthy older adults and early-stage AD, future studies should focus on EEG amplitude modulation differences between healthy controls and patients with mild cognitive impairment.

## Conclusions

This paper proposed an innovative method of quantifying resting-awake EEG spectro-temporal amplitude modulations and investigated if significant differences existed between healthy participants (NS), those diagnosed with mild symptoms of Alzheimer's disease (AD1) and patients with moderate AD (AD2). Significant differences in EEG modulations were observed for a number of electrode locations, in both comparisons (NS vs. AD1 and AD1 vs. AD2). Additionally, the proposed parameters indicated several changes in cross-frequency modulations, most notably a disappearance of delta modulations of the beta frequency band and an appearance of delta-modulations in the theta frequency band, as disease severity increased. Such findings still need to be further investigated to see if they correlate with neuropsychiatric symptoms commonly reported in the literature. Ultimately, it is hoped that the developed tool will assist clinicians with early AD diagnostics, disease severity monitoring, and objective treatment outcome measurement. Towards this end, the tool is currently being made available via the *NeuroAccelerator.org* open-source`in-the-cloud' data analysis portal.

## Author Contributions

Conceived and designed the experiments: TF FF PK RA. Performed the experiments: TF FF PK RA. Analyzed the data: TF FF. Contributed reagents/materials/analysis tools: TF FF PK RA. Wrote the paper: TF FF. Collected EEG data: PK RA. Visual selection of clean epochs: PK RA.

## References


1. Alzheimer's Association (2010) Alzheimer's disease facts and figures: 2010 report. Alzheimer's & Dementia 6: 158–194.
2. Alzheimer's Association (2012) Alzheimer's Association Report: 2012 Alzheimer's disease facts and figures. Alzheimer's & Dementia 8: 131–168.
3. Hurd MD, Martorell P, Delavande A, Mullen KJ, Langa KM (2013) Monetary costs of dementia in the United States. New England Journal of Medicine 368: 1326–1334.
4. World Health Organization and Alzheimer's Disease International (2012) Dementia: a public health priority. Technical report.
5. Leifer B (2003) Early diagnosis of Alzheimer's disease: clinical and economic benefits. J Am Geriatr Soc 51: S281–S288.
6. Terry D (1994) Neuropathological changes in Alzheimer disease. Prog Brain Res 101: 383–390.
7. Illán IA, Górriz JM, Ramírez J, Lang EW, Salas-Gonzalez D, et al. (2012) Bilateral symmetry aspects in computer-aided Alzheimer's disease diagnosis by single-photon emission-computed tomography imaging. Artificial intelligence in medicine.
8. Koch W, Teipel S, Mueller S, Benninghoff J, Wagner M, et al. (2012) Diagnostic power of default mode network resting state fMRI in the detection of Alzheimer's disease. Neurobiology of aging 33: 466–478.
9. Nordberg A, Rinne J, Kadir A, Langstrom B (2010) The use of PET in Alzheimer disease. Nat Rev Neurol 6: 78–87.
10. Schonheit B, Zarski R, Ohm T (2004) Spatial and temporal relationships between plaques and tangles in Alzheimer-pathology. Neurobiol Aging 25: 697–711.
11. Aghajani H, Zahedi E, Jalili M, Keikhosravi A, Vosoughi Vahdat B (2013) Diagnosis of Early Alzheimer's Disease based on EEG Source Localization and a Standardized Realistic Head Model. IEEE Journal of Biomedical and Health Informatics 99.
12. Adeli H, Ghosh-Dastidar S, Dadmehr N (2005) Alzheimer's disease: models of computation and analysis of EEGs. Clin EEG Neurosci 36: 131–140.
13. Kwak YT (2006) Quantitative EEG findings in different stages of Alzheimer's disease. Journal of clinical neurophysiology 23: 457–462.
14. Trambaiolli LR, Lorena AC, Fraga FJ, Kanda PA, Nitrini R, et al. (2011) Does EEG montage influence Alzheimer's disease electroclinic diagnosis? Int J Alzheimers Dis 2011: 761891.
15. Sankari Z, Adeli H, Adeli A (2012) Wavelet coherence model for diagnosis of Alzheimer disease. Clin EEG Neurosci 43: 268–78.
16. Jeong J (2004) EEG dynamics in patients with Alzheimer's disease. Clin Neurophysiol 115: 1490–1505.
17. Tsai PH, Lin C, Tsao J, Lin PF, Wang PC, et al. (2012) Empirical mode decomposition based detrended sample entropy in electroencephalography for Alzheimer's disease. J Neurosci Methods 210: 230–7.
18. Dauwels J, Srinivasan K, Ramasubba Reddy M, Musha T, Vialatte FB, et al. (2011) Slowing and loss of complexity in Alzheimer's EEG: two sides of the same coin? International journal of Alzheimer's disease 10.
19. Smit DJ, De Geus EJ, van de Nieuwenhuijzen ME, van Beijsterveldt CE, van Baal GCM, et al. (2011) Scale-free modulation of resting-state neuronal oscillations reflects prolonged brain maturation in humans. The Journal of Neuroscience 31: 13128–13136.
20. Hardstone R, Poil SS, Schiavone G, Jansen R, Nikulin VV, et al. (2012) Detrended fluctuation analysis: a scale-free view on neuronal oscillations. Frontiers in physiology 3.
21. Montez T, Poil SS, Jones BF,Manshanden I, Verbunt JP, et al. (2009) Altered temporal correlations in parietal alpha and prefrontal theta oscillations in early-stage Alzheimer disease. Proceedings of the National Academy of Sciences 106: 1614–1619.
22. Abásolo D, Hornero R, Escudero J, Espino P (2008) A study on the possible usefulness of de- trended fluctuation analysis of the electroencephalogram background activity in Alzheimer's disease. Biomedical Engineering, IEEE Transactions on 55: 2171–2179.
23. Falk TH, Fraga FJ, Trambaiolli L, Anghinah R (2012) EEG amplitude modulation analysis for semiautomated diagnosis of Alzheimers disease. EURASIP Journal on Advances in Signal Processing 2012: 1–9.
24. Trambaiolli L, Falk T, Fraga F, Lorena A, Anghinah R (2011) EEG Spectro-Temporal Modulation Energy: a New Feature for Automated Diagnosis of Alzheimer's Disease. In: Proc. Intl. Conf. IEEE EMBS. 3828–3831.
25. McKhann G, Drachman D, Folstein M, Katzman R, Price D, et al. (1984) Clinical diagnosis of Alzheimer's disease: Report of the NINCDS-ADRDA Work Group. Neurology 34: 939.
26. Association AP (2000) Diagnostic and statistical manual of mental disorders: DSM-IV-TR. American Psychiatric Publishing, Inc.
27. Brucki SM, Nitrini R, Caramelli P, Bertolucci PH, Okamoto IH (2003) Suggestions for utilization of the mini-mental state examination in Brazil. Arq Neuropsiquiatr 61: 777–81.
28. Sanei S, Chambers J (2007) EEG signal processing. Wiley-Interscience.
29. Le Van Quyen M, Foucher J, Lachaux J, Rodriguez E, Lutz A, et al. (2001) Comparison of Hilbert transform and wavelet methods for the analysis of neuronal synchrony. J Neurosci Meth 111: 83–98.
30. Buzsáki G, Draguhn A (2004) Neuronal oscillations in cortical networks. Science 304: 1926–1929.
31. Smith Z, Delgutte B, Oxenham A (2002) Chimaeric sounds reveal dichotomies in auditory perception. Nature 416: 87–90.
32. Boashash B (2003) Time Frequency Signal Analysis and Processing: A comprehensive Reference. Elsevier: Amsterdam.
33. Brenner R, Ulrich R, Spiker D, Sclabassi R, Reynolds C, et al. (1986) Computerized EEG spectral analysis in elderly normal, demented and depressed subjects. Electroenceph clin Neurophysiol 64: 483–492.
34. Coben L, Danziger W, Berg L (1983) Frequency analysis of the resting awake EEG in mild senile dementia of Alzheimer type. Electroenceph clin Neurophysiol 55: 372–380.
35. Cibils D (2002) Dementia and qEEG (Alzheimer's disease). Clin Neurophysiol 54: 289–294.
36. Babiloni C, Binetti G, Cassetta E, Dal Forno G, Del Percio C, et al. (2006) Sources of cortical rhythms change as a function of cognitive impairment in pathological aging: a multicenter study. Clinical neurophysiology 117: 252–268.
37. Schreiter-Gasser U, Gasser T, Ziegler P (1993) Quantitative EEG analysis in early onset Alzheimer's disease: a controlled study. Electroencephalography and clinical neurophysiology 86: 15–22.
38. Jarque M, Bera AK, Jarque CM, Bera AK (1987) A test for normality of observations and regression residuals. Internat Statist Rev: 163–172.
39. Deb P, Sefton M (1996) The distribution of a Lagrange multiplier test of normality. Economics Letters 51: 123–130.
40. Penttilä M, Partanen JV, Soininen H, Riekkinen P (1985) Quantitative analysis of occipital EEG in different stages of Alzheimer's disease. Electroencephalography and clinical neurophysiology 60: 1–6.
41. Huang C, Wahlund LO, Dierks T, Julin P, Winblad B, et al. (2000) Discrimination of Alzheimer's disease and mild cognitive impairment by equivalent EEG sources: a cross-sectional and longitudinal study. Clinical Neurophysiology 111: 1961–1967.
42. Putman P, van Peer J, Maimari I, van der Werff S (2010) EEG theta/beta ratio in relation to fear-modulated response-inhibition, attentional control, and affective traits. Biol Psychol 83: 73–78.
43. Robert P, Darcourt G, Koulibaly M, Clairet S, Benoit M, et al. (2006) Lack of initiative and interest in Alzheimer's disease: a single photon emission computed tomography study. European journal of neurology 13: 729–735.

44. Schutter D, Leitner C, Kenemans J, Honk J (2006) Electrophysiological correlates of cortico-subcortical interaction: A cross-frequency spectral EEG analysis. Clin Neurophysiol 117: 381–387.
45. Knyazev GG (2007) Motivation, emotion, and their inhibitory control mirrored in brain oscillations. Neuroscience and biobehavioral reviews 31: 377–395.
46. Chow TW (2000) Personality in frontal lobe disorders. Current psychiatry reports 2: 446–451.
47. Miskovic V, Ashbaugh AR, Santesso DL, McCabe RE, Antony MM, et al. (2010) Frontal brain oscillations and social anxiety: A cross-frequency spectral analysis during baseline and speech anticipation. Biological psychology 83: 125–132.
48. Schutter DJ, Knyazev GG (2012) Cross-frequency coupling of brain oscillations in studying motivation and emotion. Motivation and emotion 36: 46–54.
49. Hwang TJ,Masterman DL, Ortiz F, Fairbanks LA, Cummings JL (2004) Mild cognitive impairment is associated with characteristic neuropsychiatric symptoms. Alzheimer Disease & Associated Disorders 18: 17–21.
50. Palmer K, Berger A, Monastero R, Winblad B, Bäckman L, et al. (2007) Predictors of progression from mild cognitive impairment to Alzheimer disease. Neurology 68: 1596–1602.
51. Palva S, Palva JM (2007) New vistas for -frequency band oscillations. Trends in neurosciences 30: 150–158.
52. Wisor J, Edgar D, Yesavage J, Ryan H, McCormick C, et al. (2005) Sleep and circadian abnormalities in a transgenic mouse model of Alzheimers disease: a role for cholinergic transmission. Neuroscience 131: 375–385.
53. Jyoti A, Plano A, Riedel G, Platt B (2010) EEG, Activity, and Sleep Architecture in a Transgenic A$\beta$PP swe/PSEN1 A246E Alzheimer's Disease Mouse. Journal of Alzheimer's Disease 22: 873–887.
54. Braak H, Braak E, Kalus P (1989) Alzheimer's disease: areal and laminar pathology in the occipital isocortex. Acta neuropathologica 77: 494–506.
55. Payami H, Zareparsi S, Montee K, Sexton G, Kaye J, et al. (1996) Gender difference in apolipoprotein E-associated risk for familial Alzheimer disease: a possible clue to the higher incidence of Alzheimer disease in women. Am J Hum Genet 58: 803.
56. Herrera EJ, Caramelli P, Silveira ASB, Nitrini R (2002) Epidemiologic survey of dementia in a community-dwelling Brazilian population. Alzheimer Dis Assoc Disord 16: 103–108.
57. Ott A, Breteler MM, van Harskamp F, Claus JJ, van der Cammen TJ, et al. (1995) Prevalence of Alzheimer's disease and vascular dementia: association with education. The Rotterdam study. BMJ (Clinical research ed) 310: 970–973.
58. Lopes MA, Ferrioli E, Nakano EY, Litvoc J, Bottino CMC (2012) High Prevalence of Dementia in a Community-Based Survey of Older People from Brazil: Association with Intellectual Activity Rather than Education. Journal of Alzheimer's Disease 32: 307–316.
